# Supplementary material for: North Atlantic ventilation change over the past three decades is potentially driven by climate change
Source: Nat Commun. 2026 Jan 7;17:200. doi: 10.1038/s41467-025-67923-x (PMC12780181; doi:10.1038/s41467-025-67923-x)
Supplement: Supplementary file 1 — Supplementary Information [file 41467_2025_67923_MOESM1_ESM.pdf]

Supplementary Information for "North Atlantic ventilation change over the past three decades is potentially driven by climate change"

Haichao Guo[1,2,*], Wolfgang Koeve[1], Iris Kriest[1], Ivy Frenger[1], Toste Tanhua[1], Peter Brandt[1,3], Yanchun He[4], Tianfei Xue[1], and Andreas Oschlies[1,3]

[1]GEOMAR Helmholtz Centre for Ocean Research Kiel, Kiel, Germany
[2]Department of Oceanography, School of Ocean and Earth Science and Technology, University of Hawaii at Mānoa, Honolulu, USA
[3]Kiel University, Kiel, Germany
[4]Nansen Environmental and Remote Sensing Center, Bjerknes Centre for Climate Research, Bergen, Norway

[*]Corresponding author: hguo@geomar.de

Contents of this file

1. Fig. S1 to S8
2. Tab. S1

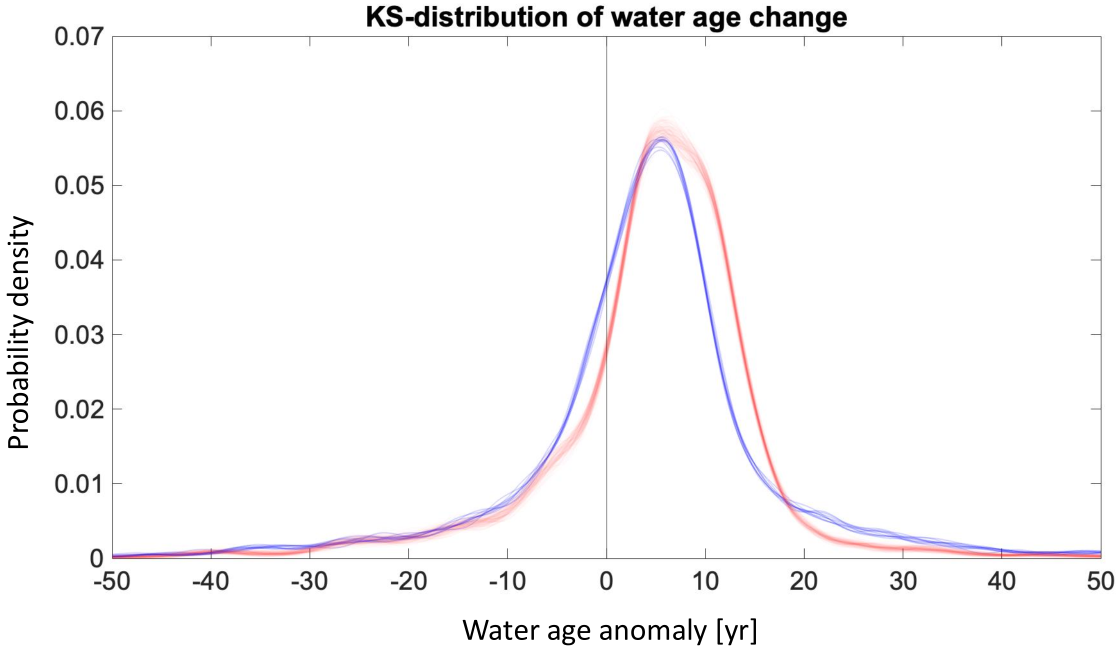

Fig. S1: Kernel density estimates of the change in water age (Δage, in yr) in the North Atlantic: from the 1990s to the 2000s (blue) and from the 2000s to the 2010s (red). Random measurement uncertainties within 3% have been incorporated. The plots are based on 100 Monte Carlo simulations.

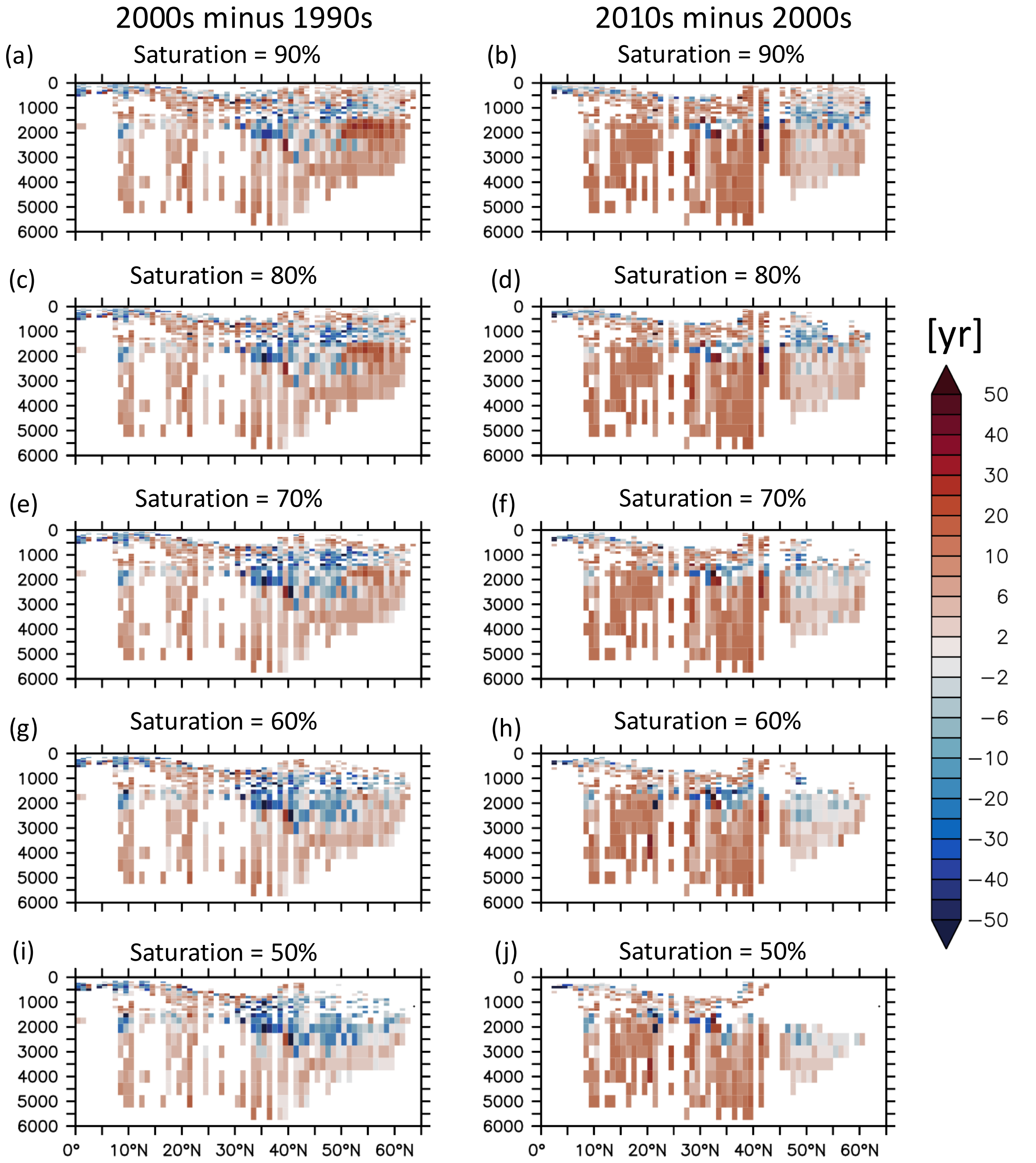

Fig. S2: The change in water age (Δage, in yr) in the North Atlantic comparing data from the 1990s to the 2000s (a,c,e,g,i) and from the 2000s to the 2010s (b,d,f,h,j). Different assumptions about saturation levels are applied in the IG-TTD calculations of the individual panels.

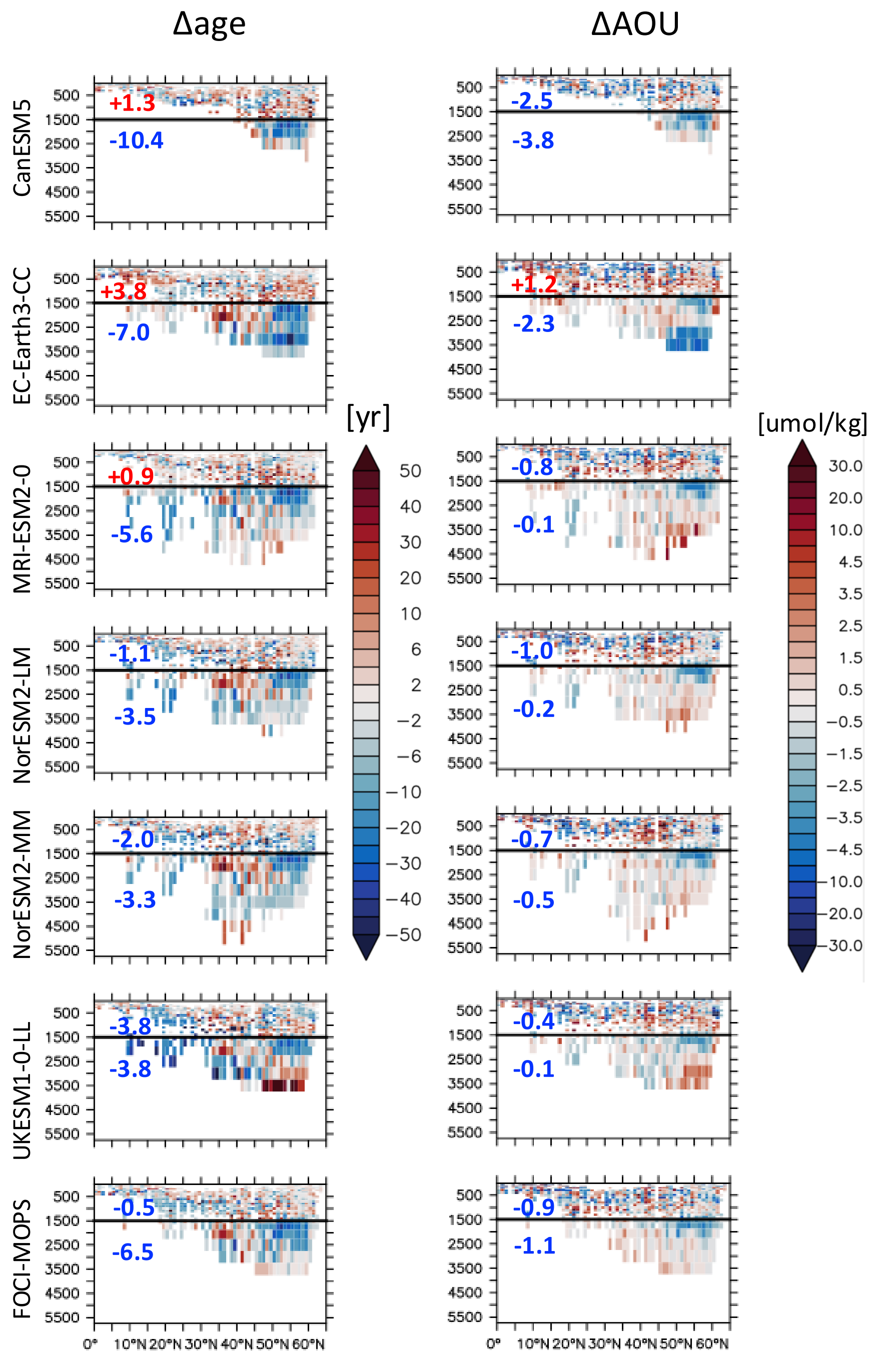

Fig. S3: Differences in change of age (Δage in yr, left panels) and change of apparent oxygen utilization (ΔAOU, μmol/kg, right panels) between models and observations (model minus observation). We used Δage and ΔAOU from the 1990s to the 2000s. Black lines separate the upper 1500 m from deeper layers. Numbers indicate the mean Δage and ΔAOU differences, with colors denoting whether models underestimate (blue) or overestimate (red) the aging signal.

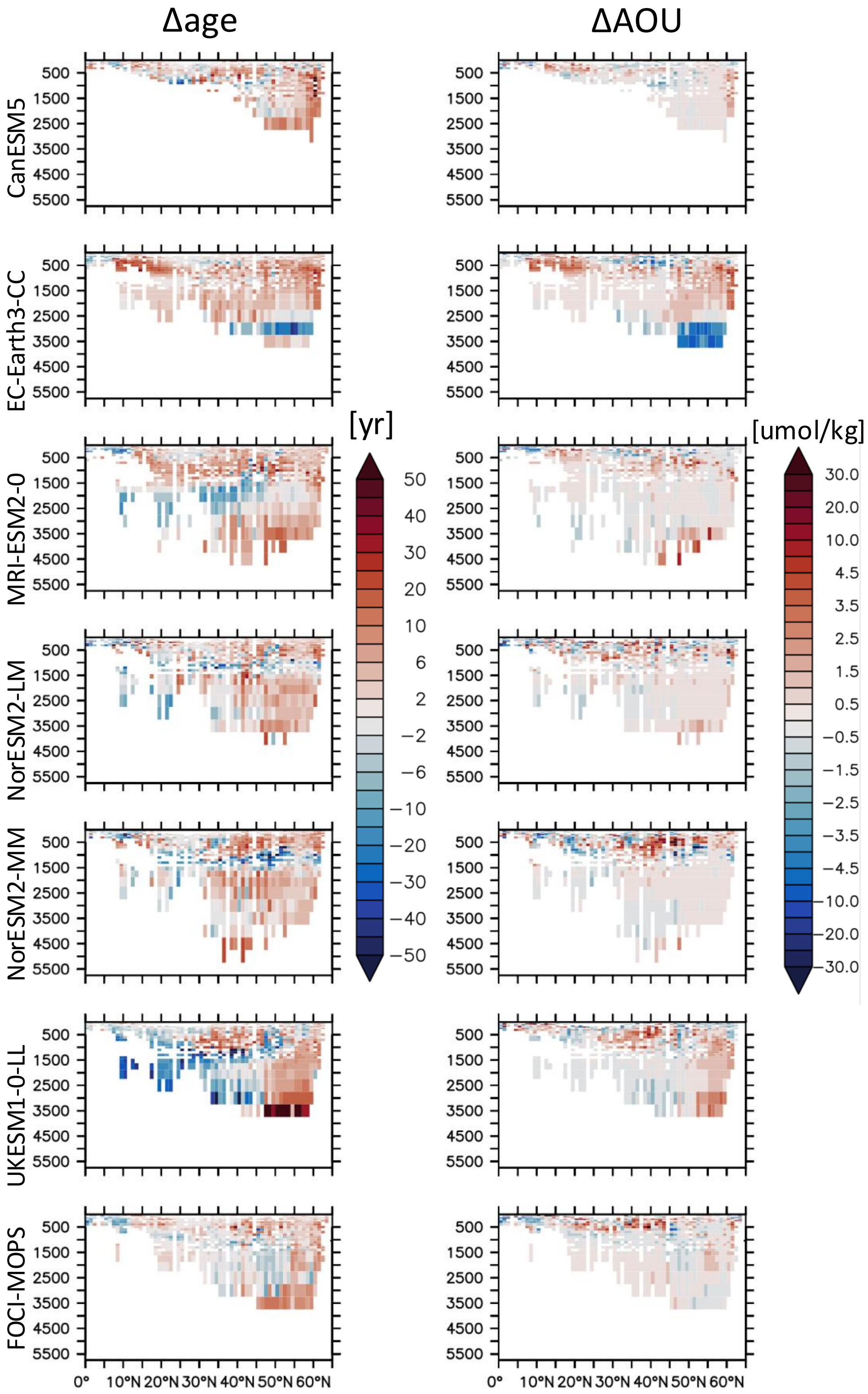

Fig. S4: Left and right panels present the zonal average for Δage (in units of years) and ΔAOU (in units of μmol/kg) in the North Atlantic from the 1990s to the 2000s across seven Earth System models.

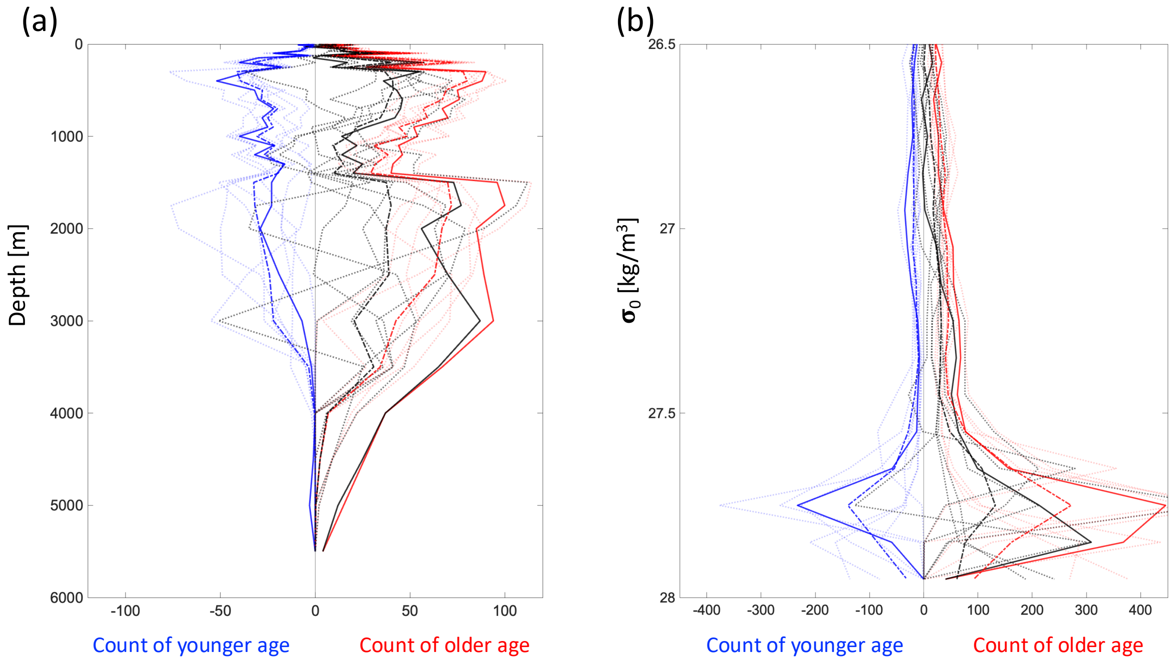

Fig. S5: Number of boxes indicating older (red) and younger (blue) water age and their difference (black=red minus blue) from the 1990s to the 2000s, plotted across (a) depths and (b) isopycnals. The solid lines depict the GLODAPv2.2022 data, while the dashed lines represent the multi-model mean. The dotted lines illustrate individual model results.

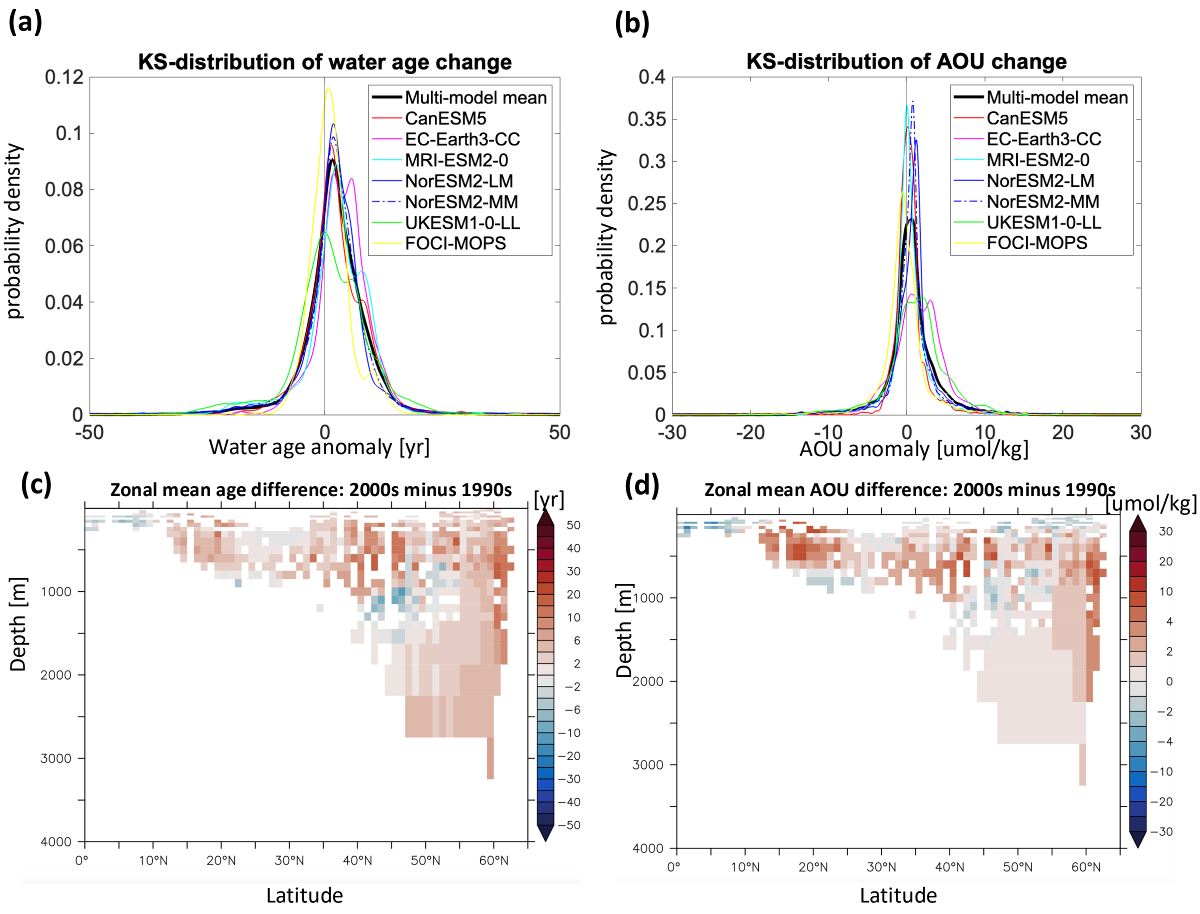

Fig. S6: Simulated temporal changes in water age and apparent oxygen utilization (AOU) in the North Atlantic from the 1990s to the 2000s. (a, b) display the Kernel Smoothing density estimation for the changes in water age (Δage, years) and AOU (ΔAOU, μmol/kg) in the North Atlantic, comparing data from the 1990s to the 2000s across seven individual models and the model average. (c, d) present the multi-model mean of the zonal average for Δage and ΔAOU in the North Atlantic. Here we did not subsample the models' outputs according to when the observations were available.

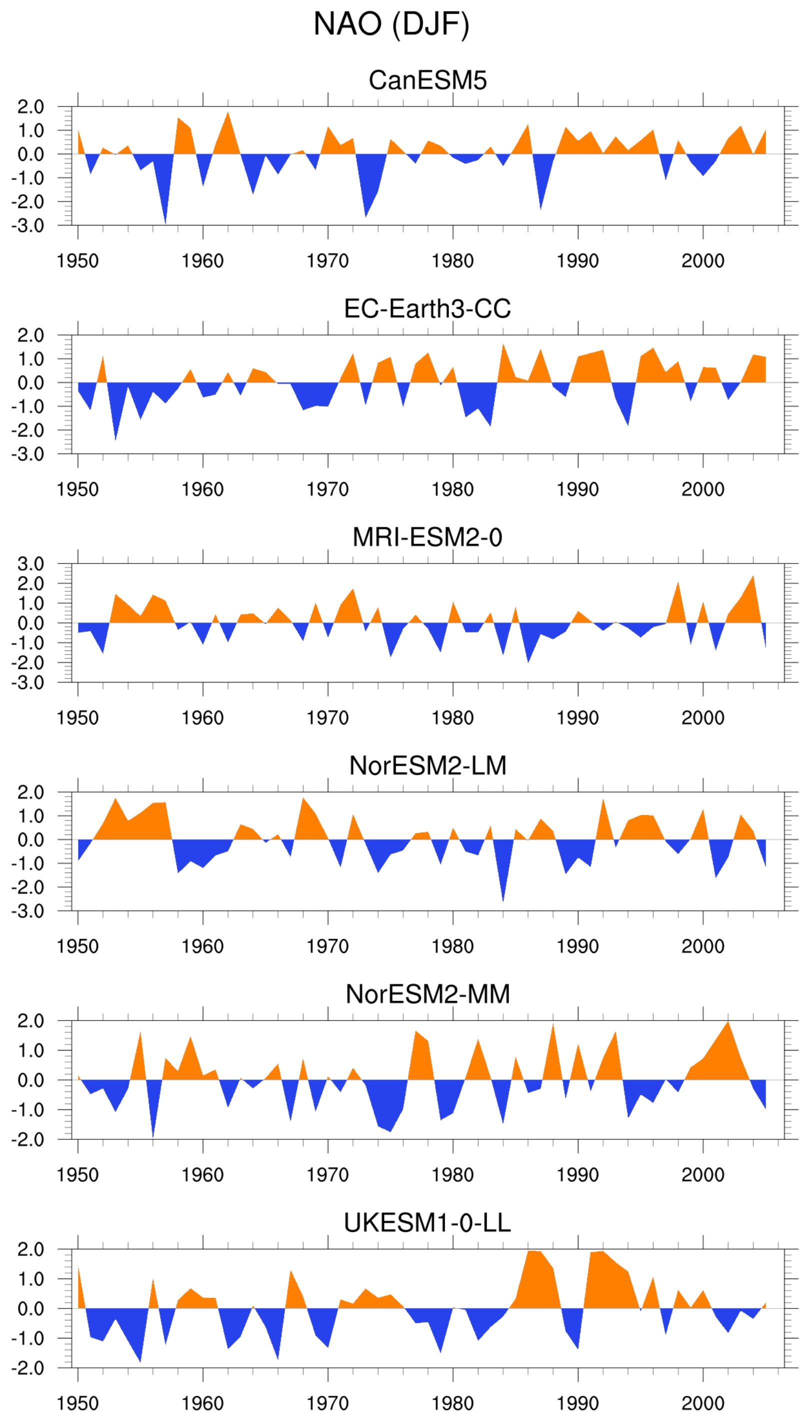

Fig. S7: The North Atlantic Oscillation (NAO) index in individual models. The NAO index is defined as the principal component of the dominant Empirical Orthogonal Function (EOF) pattern of winter (DJF: December-February) sea-level pressure over the North Atlantic region (20°N–80°N, 90°W–40°E). The phase of the NAO index varies among models, indicating different internal variabilities of the used climate models.

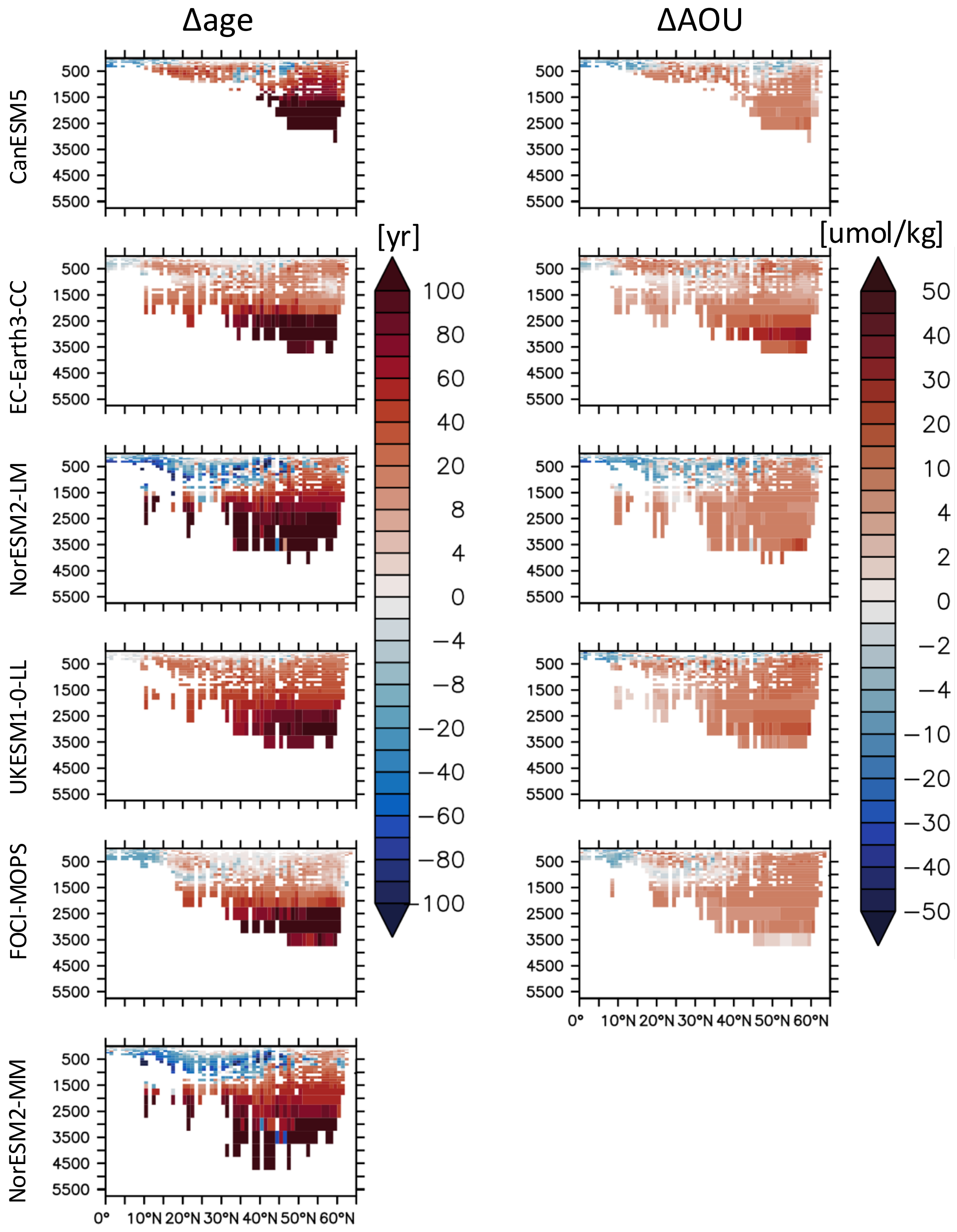

Fig. S8: Left and right panels present the zonal average for Δage (in units of years) and ΔAOU (in units of mmol/kg) in the North Atlantic from the 2010s to the 2090s across Earth System models. The simulations are under high carbon dioxide emission scenario (SSP5-8.5). Note that, ideal age data in MRI-ESM-2-0 and dissolved oxygen concentration data in NorESM2-MM were not accessible for the SSP5-8.5 scenario during the course of our study (accessed in August of 2025, `https://esgf-metagrid.cloud.dkrz.de/search/cmip6-dkrz/`).

Tab. S1: Temporal change (2000s minus 1990s) of North Atlantic water age and Apparent Oxygen Utilization (AOU) across multi-model mean and seven individual Earth System models. Here the models' outputs are not subsampled according to when the observations were available.

Variable	Models	Mean	Median	Mode	Volume weighted mean
ΔWater age (yr)	Multi-model mean[a]	+2.2 ± 0.9	+2.2 ± 1.0	+1.5 ± 0.8	+2.4 ± 1.6
	CanESM5	+3.1	+2.1	+1.4	+3.6
	EC-Earth3-CC	+3.9	+3.9	+1.9	+3.8
	MRI-ESM2-0	+2.7	+3.0	+2.5	+2.4
	NorESM2-LM	+1.9	+2.2	+1.9	+3.2
	NorESM2-MM	+1.6	+2.2	+1.8	+3.0
	UKESM1-0-LL	+1.2	+1.1	-0.1	-1.2
	FOCI-MOPS	+1.3	+0.9	+0.8	+2.0
ΔAOU (μmol/kg)	Multi-model mean[a]	+0.7 ± 0.5	+0.6 ± 0.6	+0.6 ± 0.8	+0.6 ± 0.2
	CanESM5	+0.4	+0.2	+0.1	+0.5
	EC-Earth3-CC	+1.4	+1.4	+0.7	+0.9
	MRI-ESM2-0	+0.4	+0.2	+0.0	+0.6
	NorESM2-LM	+0.8	+1.0	+1.2	+0.9
	NorESM2-MM	+0.7	+0.7	+0.8	+0.4
	UKESM1-0-LL	+1.2	+1.3	+2.1	+0.7
	FOCI-MOPS	-0.3	-0.4	-0.5	+0.3

[a] For multi-models mean: mean ± one standard deviation
